# Similarities and differences between three different types of white dot syndrome and the therapeutic possibilities

**Published:** 2018

**Authors:** Roxana Cozubas, Emil Ungureanu, Sinziana Luminita Instrate, Cristina Alexandrescu, Razvan Vladimir Nanu, Laura Carstocea, Liliana Mary Voinea, Radu Ciuluvica

**Affiliations:** * ”Grigore Alexandrescu”, Emergency Hospital for Children, Bucharest, Romania; ** Ophthalmology Department, University Emergency Hospital; “Carol Davila” University of Medicine and Pharmacy, Bucharest, Romania; *** “Sf. Ioan” Emergency Hospital, Bucharest, Romania; **** PhD Student, “Carol Davila” University of Medicine and Pharmacy, Bucharest, Romania; ***** Anatomy Department, “Carol Davila” University of Medicine and Pharmacy, Bucharest, Romania

**Keywords:** white dot syndrome, choroid, uveitis

## Abstract

White dot syndromes consists a group of inflammatory eye diseases with an unknown etiology involving the external retina, retinal pigment epithelium, choroid or combinations of them. They affect one or both eyes, at the same time or not.

White dot syndromes are often self-limited, with a variable prognosis, depending on the type of the disease. The purpose of this article was to look at the similarities and the differences between the different types of syndromes and the therapeutic possibilities existing at present.

**Abbreviations:** WDS = White dot syndromes, MEWDS = Multiple Evanescent White Dot Syndrome, APMPPE = Acute Posterior Multifocal Placoid Pigment epitheliopathy, SC = serpiginous choroiditis RPE = retinal pigment epithelium

The WDS consists of a group of inflammatory eye diseases of an unknown cause that involves external retina, RPE or the choroid, or combinations of those, affecting just one or the both eyes [**1**]. Symptoms associated with WDS include blurred vision, loss of visual field (blind spot enlargement), photopsia, idiopathic and myodesopsia [**2**]. Usually it occurs in patients over 20-60 years of age, with the exception of Birdshot chorioretinopathy or seroprevalent choroiditis that affects patients aged 20 and 30 years old. MEWDS, Birdshot chorioretinopathy, and multifocal choroiditis with panuveitis mainly occur in women [**3**].

WDS are linked to the inflammatory cells situated in the anterior chamber of the eye and vitreous [**1**]. White spots are subtle or prominent, depending on the disease. The causes of these syndromes are unknown, and serological evaluation is often negative. It is usually self-limited, with variable prognosis depending on the disease [**4**].

There are a sum of multiple diseases that are affecting the layers of the retina and the choroid (in multifocal points), which are often connected with the whitening of the retina in some parts, and which have unknown etiology. These entities are now known as WDS or, by a former name of “multifocal inflammatory chorioretinopathies” [**3**].

Classically, WDS includes multifocal choroiditis and panuveitis, APMPPE, SC, MEWDS, Birdshot Chorioretinopathy and Diffuse Unilateral Subacute Neuroretinitis (DUSN) [**2**]. 

There are also other conditions presenting with white dots at fundus examination, and those include progressive subretinal fibrosis and uveitis syndrome, acute retinal pigment epitheliitis, punctate inner choroidopathy (PIC), acute zonal occult outer retinopathy (AZOOR), acute macular neuroretinopathy, unilateral acute idiopathic maculopathy. Recently, other entities were included in this group - relentless placoid chorioretinitis and unifocal helioid choroiditis [**2**].

**Acute Posterior Multifocal Placoid Pigment epitheliopathy (APMPPE)**

**Introduction**

APMPPE is a very rare and idiopathic inflammatory disease which affects young adults between the ages of 20 and 50 years. Regarding the evolution, the disease is self-limiting with a spontaneous healing seen in some of the patients and with a good prognosis, even if we consider the absence of the treatment. At the onset, the disease is associated with altered general condition, many flu-like symptoms, hearing loss, possibly nodal erythema, tinnitus, after that followed by a small decrease on the visual acuity, initially observed just in one eye, but soon followed by a few days also in the other eye [**5**].

**Epidemiology and pathogenesis**

The etiology is unknown and there are at least two possible mechanisms that explain this condition. APMPPE was considered an inflammatory process that begins either at the RPE level or in the small coronary artery with vasculitis and ischemic changes, associated systemic vasculitis resulting in a diffuse vasculitis process interrupting coronary perfusion and appearance of ocular characteristics [**6**]. In APMPPE, choroid ischemia leads to a disruption of the RPE barrier. Another proposed mechanism is a delayed hypersensitivity reaction with the activation of T lymphocytes sensitized to an unknown agent resulting in occlusive vasculitis at the choroidal level [**7**].

**Treatment**

Because the disease is generally an autoimmune pathology, we don’t have a reason for treatment, which is conducted only in the case if the lesions involve the fovea or if neurological complications are encountered. In most of the cases, we find a gradual improvement of the visual acuity, but this happens over several months; by that time, patients will obtain visual acuity of at least 2/3. Steroids are proven to provide a theoretical advantage in shortening the length of the disease or in modifying effects of the central vision [**5**,**8**].

**Multiple Evanescent White Dot Syndrome (MEWDS)**

**Introduction**

MEWDS is a rare disorder, in which we observe unilateral evolution; it affects young women in the decades 3 or 4 of life, usually with a history of viral infections in the past. Evolution is normally limited, with a good prognostic and a spontaneous healing beginning at a few weeks and ending at a few months. We found that a small number of patients described a chronic bilateral evolution with multiple recurrences followed by choroidal neovascularization [**9**]. At the beginning, patients describe an acute decrease in visual acuity accompanied by scotoma and sometimes by photopsia. At the objective exam, a very much discrete inflammation of the vitreous and multiple white-yellowish deep-distributed concentric stains around the macula were detected. Patients diagnosed with MEWDS can show an edema at the level of optic nerve, which determines a reaming type defect at the level of blind spot into the visual field. Initially, the disease debuts as a flu seen in almost half of the patients. The disease can be typical unilateral, but we also found bilateral cases in the literature [**10**]. 

**Eye manifestations**

Patients have sudden loss of sight, unilaterally. Bilateral cases have also been reported, but tend to be asymmetrical [**11**]. Patients complain about scotoma and photopsia, especially at the level of temporal visual field. One third of the cases may occur in a viral prodrome. Visual acuity is variable and stretches from 1 to 1/20. There may be found a small myopia and also a pupillary afferent defect [**12**]. The anterior segment seems normal, while at the level of posterior pole we can find vitritis (of various intensities), edema, or hyperemia of the optical disc and, typically, multiple white points (poorly defined) at the RPE level or in the deep retina, in the posterior pole, may appear. The lesions are pale in color in the first few weeks of the disease [**13**].

The retinal vessels may also present a lining; a yellow-orange granular appearance, which is characteristic, at the level of fovea. It may be present acutely, but fovea does not come back to a normal appearance. Choroidal scars that resemble a multifocal choroiditis may develop in some patients, especially in those with chronic diseases [**14**].

**Diagnosis**

Regarding the diagnosis, it is established based only on the ocular type of manifestations, in this disease. Visual field can record a reaming at the level of the blind spot. We can find temporal or paracentral scotoma in the visual field [**12**]. 

**Treatment**

Because MEWDS has a limited evolution, no treatment is required. We found a study that described the favorable clinical results in conjunction with the treatment including intravitreal ranibizumab for subfoveal choroidal neovascularization associated with the MEWDS syndrome; this action made possible a significant reduction at the level of macular thickness after only two injections of Anti-VEGF agent [**15**-**17**].

**Serpiginous choroiditis (SC)**

**Introduction**

SC is a very rare disease, with recurring evolution, usually bilateral, although sometimes asymmetrical between the two eyes. Affected patients could present a debut in the ages from 30 to 60 years and may have a reserved prognosis [**18**]. These patients are complaining about a blurred vision, some metamorphopsia and the characteristic appearance resembling a central or a paracentral scotoma. Regarding the ocular signs, we can see a subretinal infiltrate at the level of the posterior pole, together with stress margins located initially around the papilla; this structure tends to expand progressively beginning from the optic nerve and continuing to the macula in a pattern of a serpentine. We can observe also the old lesions that are atrophy of pigmentary epithelium, seen in the same eye, that can also alternate with newly formed lesions that present a hyperplasia of RPE [**19**]. 

**Epidemiology and Pathogenicity**

SC was described under several names, including helicoid or geographic choroidopathy. The disease has a chronic, progressive evolution and apparently affects healthy patients in decade 2-7, with equal damage to the sexes. The appellants are a rule and can occur weeks to years after the initial event. Although it is bilateral, it can affect asymmetrical eyes [**20**]. 

The etiology is not not until now fully known and understood: a possible immune-mediated mechanism has been proposed by time, linked to the increase in the finding of the HLA-B7 antigen and together with the mixing of retinal antigen. Also, an association with the varicella-zoster virus was reported, although, at this time, it is not significant [**21**].

**Symptoms**

Patients who have SC present subjective central or paracentral scotoma, with loss of visual acuity, as well as metamorphopsia or loss of visual field. Some lesions may be asymptomatic until fovea’s involvement [**22**]. 

**Diagnosis**

The diagnosis is determined after the typical occurrence of the lesions. Fluorescein angiography shows early hypofluorescence and delayed hyperfluorescence in the case of active lesions. Indocyanine green angiography shows hypofluorescence in the case of active lesions (secondary to inflammatory obstructions of choriocapillaris or fluorescent blocked by swallowed RPE). Active lesions show diffuse pigment loss, choroidal vessels and delayed staining at angiofluorography [**23**]. 

Retinal vascular staining may occur in adjacent regions to active lesions. Associated choroidal neovascularization shows leaks that often come from the edge of old scars [**24**].

**Treatment**

Based on the presumed factor that led to the choroiditis, the main approach is to use immunosuppressive agents. Initial high-dose corticosteroids usually limit acute choroiditis, but long-term treatment needs to prevent recurrences, based on the immunomodulatory agents. Some clinical studies have proposed the initial treatment with corticosteroids without immunomodulatory agents [**25**], but others are pro initiating treatment with a combination of corticosteroids and immunomodulatory agents, which results in a better long-term control [**26**].

Local treatment is made with topical administration of corticosteroid, but this may found to be necessary in patients who have anterior segment inflammation. Intravenous, peribulbar, subtenon, and intravitreal corticosteroid injections they were all also been used with variable success [**27**].

## Conclusion

WDS are a list of disorders affecting the choroid, retina, and retinal pigment epithelium. They come with a diagnostic which can be significant and with some therapeutic challenges for the clinician, but also for the research scientist. Observation of signs together with a careful documentation of findings, long follow-up of cases, and an appropriate treatment of reactivation, are needed for a successful management of white dot cases. As far as the treatment is concerned, options today are related to steroid and immunosuppressive use – **Table 1**.

**Table 1:** Comparison between three different types of WDS

Disease	Presentation and debut	Ocular findings	White dot description	Treatment
AMPPE	Young male, but also female patients (in their 20s-30s) A prodromal flu-like illness may be present Can be bilateral Scotoma Photopsia Acute debut Blurred vision	Mild anterior and posterior cells Early hypofluorescence with a late staining on the fluorescein angiography	Multifocal flat, gray-white lesions which forms a placoid appearance; they are found at the level of posterior pole, in RPE; the lesions are improving within 1-2 weeks and patients may have disc swelling	Observation but also with Corticosteroids
MEWDS	Young females (in their 20s up to 50s) Usually unilateral Prodromal flu-like illness Decreased vision Photopsias Scotoma	Mild vitreous cells At the fluorescein angiography we observe a hypo-fluorescence Decreased a wave on the ERG Most patients will obtain a full recovery	Orange specks at the level of the fovea Also numerous small white spots, seen throughout mid but also peripheral retina	Observation
SC	Rare enitity Patients are older, usually male (in their 30’s to 60’s) No prodromal illness Bilateral	The visual prognosis is poor Mild or even absent vitreous cells A percent of 33 develop CNV In fluorescein angiography, we detect early hypo- and late hyper-fluorescence	The lesions are situated peripapillary and are coloured yellow/ grey; they are contiguous large placoid and spreading out centrifugal to peripheral retina	Immunosuppression, sometimes antivirals, Some authors also take photocoagulation for CNV in consideration

This chart was produced for the purpose of the article, using the information found in Gass [**28**].

**Conflict of interests**

The authors declare no conflict of interest.

**Acknowledgements**

All authors have equal contribution.
